# Supplementary material for: COVID-19 Vaccination Booster Dose: Knowledge, Practices, and Intention among Pregnant/Planning to Get Pregnant and Lactating Women
Source: Vaccines (Basel). 2023 Jul 17;11(7):1249. doi: 10.3390/vaccines11071249 (PMC10383860; doi:10.3390/vaccines11071249)
Supplement: Supplementary file 1 [file vaccines-11-01249-s001.zip › vaccines-2473628-supplementary.pdf]

Supplementary Materials:

S1: COVID-19 booster dose intentions in pregnant/planning to get pregnant and lactating women questionnaire.

- Are you?

Planning to get pregnant.

Lactating.

Pregnant.

- To which age group do you belong?

18-29 years old

30-39 years old

≥ 40 years old

- What is your educational level?

High school or less

Diploma or higher

- What is your household monthly income level?

Less than 500 JOD

500-1000 JOD

More than 1000 JOD

- Are you a smoker?

No

Yes

- Do you have any chronic diseases?

No

Yes

Participants' experiences toward COVID-19.

- Did you get infected with COVID-19?

No

Not sure

Yes

- Do you personally know someone who died due to COVID-19?

No

Yes

Participants' attitudes toward COVID-19.

- In your opinion, what is the likelihood that you will be infected with COVID-19 during the next 6 months?

I don't think I will be infected.
I think I will be infected with mild symptoms.
I think I will be infected with severe symptoms.

- In your opinion, how serious is the infection with COVID?

1(low risk) - 5(high risk)

<u>Participants' experiences toward COVID-19 vaccine.</u>

- What was your reasons for receiving COVID-19 vaccines?

Imposed laws.
Conviction and imposed laws.
Conviction.

- What was the vaccine type?

Pfizer
Astra Zeneca
Sinopharm

- In your opinion, how severe were your side effect due to COVID-19 vaccine?

No symptoms
Mild
Moderate
Severe

- Which of the following side effect level you experienced due to COVID-19 vaccine?

Symptoms	Yes	No
Fever		
Chills		
Diarrhea		
Cough		
Otitis media		
Loss of smell and taste senses		
No symptoms		

<u>Knowledge of COVID-19.</u>

<u>Knowledge about the symptoms of COVID19</u>

- What are the symptoms of COVID19?

Symptoms	Yes	No
Fever		
Chills		

Diarrhea		
Cough		
Otitis media^		
Loss of smell and taste senses		
No symptoms		

^ False statement

Knowledge about the protective practices against COVID19

- Which of the following practices may prevent COVID-19 infection?

Procedures	Yes	No
Washing hands with regular soap		
Using detergents		
Social distancing		
Avoid touching face/mouth/nose/eyes		
Avoid consuming meat ^		
Medical herb consumption ^		

^ False statement

Knowledge about the transmission of COVID19

- How COVID19 can be transmitted?

	Yes	No
Drinking unclean water^		
Eating unclean food^		
Inhalation of respiratory droplets of infected person		
Eating or touching wild animals^		

Knowledge about the management of COVID19

- Is there currently a drug in the pharmacies and hospitals that cure COVID-19 completely?

Yes
No

- What is the protective time that the vaccine provides against COVID-19?

Days
Months
Years

- Is it necessary for the booster dose of the vaccine to be of the same type of vaccine used in the initial doses?

Yes
No

- Which people need the booster dose?

Children
Elderly
All people

Adherence to protective practices against COVID-19

- Which of the following practices have you taken to protect yourself from COVID19?

	Always	Usually	Sometimes	Rarely	Never
Wearing face masks					
Washing hands with regular soap					
Using detergents					
Social distancing					
Avoid touching face/mouth/nose/eyes					

Intention toward receiving COVID-19 booster dose.

- Do you intend to receive the booster dose for COVID-19 vaccine?

No
Maybe
Yes

- If you answered "No" or "Not sure" regarding the intent to receive a COVID-19 booster dose which of the following is your reason? (You can choose more than one)

	Yes	No
The booster dose will not provide me with any further protection against COVID-19.		
The booster dose will have severe side effects.		
I can't tolerate another dose because the side-effects of the previous ones were severe.		
I was infected with COVID-19, therefore I do not need the booster dose.		
The benefits of a booster dose have not been scientifically proven		
The booster dose is a conspiracy to boost corporate profits.		
I am worried that the booster dose will harm my fetus/infant		
Lack of sufficient studies that evaluated the safety of COVID-19 on pregnant/lactating women		
The vaccine may infect the fetus/infant with COVID-19		

Table S1. Variable association with knowledge and practice.

		Knowledge			Practice		
		Low	High		Low	High	
		**Frequency (%), or median (95% Cl.)**	**Frequency (%), or median (95% Cl.)**	**p-value**	**Frequency (%), or median (95% Cl.)**	**Frequency (%), or median (95% Cl.)**	**p-value**
Age	18-29 years old	279 (71.5%)	157 (51.5%)	<0.001	231 (63.8%)	205 (61.6%)	0.540
	30-39 years old	111 (28.5%)	148 (48.5%)		131 (36.2%)	128 (38.4%)	
Pregnancy status	Planning to get pregnant	181 (46.4%)	174 (57%)		179 (49.4%)	176 (52.9%)	
	Lactating	96 (24.6%)	42 (13.8%)	<0.001	75 (20.7%)	63 (18.9%)	0.660
	Pregnant	113 (29%)	89 (29.2%)		108 (29.8%)	94 (28.2%)	
Education	Low	155 (39.7%)	37 (12.1%)	<0.001	110 (30.4%)	82 (24.6%)	0.090
	High	235 (60.3%)	268 (87.9%)		252 (69.6%)	251 (75.4%)	
Household monthly income level	< 500 JOD	190 (48.7%)	52 (17%)		131 (36.2%)	111 (33.3%)	
	500-1000 JOD	150 (38.5%)	150 (49.2%)		149 (41.2%)	151 (45.3%)	
	> 1000 JOD	50 (12.8%)	103 (33.8%)	<0.001	82 (22.7%)	71 (21.3%)	0.535
Do you have any chronic diseases?	No	367 (94.1%)	271 (88.9%)	0.012	334 (92.3%)	304 (91.3%)	0.640
	Yes	23 (5.9%)	34 (11.1%)		28 (7.7%)	29 (8.7%)	
Smoking	No	327 (83.8%)	250 (82%)	0.513	295 (81.5%)	282 (84.7%)	0.263
	Yes	63 (16.2%)	55 (18%)		67 (18.5%)	51 (15.3%)	
In your opinion, how dangerous is the COVID infection ?		3 (3-4)	4 (4-5)	<0.001	3 (3-4)	4 (4-5)	<0.001
Previously infected with COVID-19	No	126 (32.3%)	90 (29.5%)		101 (27.9%)	115 (34.5%)	
	Not sure	82 (21%)	47 (15.4%)	0.057	71 (19.6%)	58 (17.4%)	0.167
	Yes	182 (46.7%)	168 (55.1%)		190 (52.5%)	160 (48%)	
Perceived possibility of becoming infected with COVID-19 in the next 6 months	I don't think I will be infected	160 (41%)	89 (29.2%)		125 (34.5%)	124 (37.2%)	
	I think I will be infected with mild symptoms	211 (54.1%)	208 (68.2%)	<0.001	221 (61%)	198 (59.5%)	0.611
	I think I will be infected with severe symptoms	19 (4.9%)	8 (2.6%)		16 (4.4%)	11 (3.3%)	
Do you personally know someone who died due to COVID-19	No	88 (22.7%)	66 (21.6%)		85 (23.5%)	69 (20.8%)	
	Yes	299 (77.3%)	239 (78.4%)	0.730	276 (76.5%)	262 (79.2%)	0.394

Knowledge level	Low	.	.	208 (57.5%)	182 (54.7%)	0.457
	High	.	.	154 (42.5%)	151 (45.3%)	

Table S2. Variables association with the intention to get a booster dose of COVID-19.

		No	Maybe	Yes	
		**Frequency (%), or median (95% Cl.)**	**Frequency (%), or median (95% Cl.)**	**Frequency (%), or median (95% Cl.)**	**p-value**
Age	18-29 years old	85 (70.2%)	111 (68.1%)	240 (58.4%)	0.016
	30-39 years old	36 (29.8%)	52 (31.9%)	171 (41.6%)	
Pregnancy status	Planning to get pregnant	57 (47.1%)	75 (46%)	223 (54.3%)	0.006
	Lactating	32 (26.4%)	44 (27%)	62 (15.1%)	
	Pregnant	32 (26.4%)	44 (27%)	126 (30.7%)	
Education	Low	37 (30.6%)	58 (35.6%)	97 (23.6%)	0.011
	High	84 (69.4%)	105 (64.4%)	314 (76.4%)	
Household monthly income level	< 500 JOD	55 (45.5%)	57 (35%)	130 (31.6%)	0.003
	500-1000 JOD	49 (40.5%)	79 (48.5%)	172 (41.8%)	
	> 1000 JOD	17 (14%)	27 (16.6%)	109 (26.5%)	
Do you have any chronic diseases?	No	111 (91.7%)	153 (93.9%)	374 (91%)	0.529
	Yes	10 (8.3%)	10 (6.1%)	37 (9%)	
Smoker	No	106 (87.6%)	151 (92.6%)	320 (77.9%)	<0.001
	Yes	15 (12.4%)	12 (7.4%)	91 (22.1%)	
Vaccine type	Pfizer	95 (78.5%)	129 (79.1%)	296 (72%)	0.002
	Astra Zeneca	5 (4.1%)	10 (6.1%)	62 (15.1%)	
	Sinopharm	21 (17.4%)	24 (14.7%)	53 (12.9%)	
Side effect level	No symptoms	21 (17.4%)	21 (12.9%)	41 (10%)	0.156
	Mild	41 (33.9%)	56 (34.4%)	135 (32.8%)	
	Moderate	44 (36.4%)	71 (43.6%)	170 (41.4%)	
	Severe	15 (12.4%)	15 (9.2%)	65 (15.8%)	
Reasons for receiving COVID-19 vaccines	Imposed laws	59 (49.2%)	32 (20%)	19 (4.6%)	<0.001
	Conviction and imposed laws	19 (15.8%)	37 (23.1%)	56 (13.7%)	
	Conviction	42 (35%)	91 (56.9%)	334 (81.7%)	
Do you personally know someone who died due to COVID-19?	No	35 (29.2%)	26 (16%)	93 (22.7%)	0.029
	Yes	85 (70.8%)	137 (84%)	316 (77.3%)	
	No	47 (38.8%)	48 (29.4%)	121 (29.4%)	0.167

Previously infected with COVID-19	Not sure	17 (14%)	37 (22.7%)	75 (18.2%)	
	Yes	57 (47.1%)	78 (47.9%)	215 (52.3%)	
Perceived possibility of becoming infected with COVID-19 in the next 6 months	I don't think I will be infected	63 (52.1%)	70 (42.9%)	116 (28.2%)	<0.001
	I think I will be infected with mild symptoms	56 (46.3%)	85 (52.1%)	278 (67.6%)	
	I think I will be infected with severe symptoms	2 (1.7%)	8 (4.9%)	17 (4.1%)	
In your opinion, how dangerous is the COVID infection ?		3 (3-4)	3 (3-4)	3 (3-4)	<0.001
Knowledge level	Low	86 (71.1%)	107 (65.6%)	197 (47.9%)	<0.001
	High	35 (28.9%)	56 (34.4%)	214 (52.1%)	
Practice Level	Low	70 (57.9%)	108 (66.3%)	184 (44.8%)	<0.001
	High	51 (42.1%)	55 (33.7%)	227 (55.2%)	

Table S3. Reasons for booster dose refusal/hesitancy.

**Reasons**	**Frequency (%)**
The booster dose will not provide me with any further protection against COVID-19.	178 62.7%
The booster dose will have severe side effects.	213 75.0%
I can’t tolerate another dose because the side-effects of the previous ones were severe.	147 51.8%
I was infected with COVID-19, therefore I do not need the booster dose.	102 35.9%
The benefits of a booster dose have not been scientifically proven	180 63.4%

The booster dose is a conspiracy to boost corporate profits.	202<br>71.1%
I am worried that the booster dose will harm my fetus/infant	258<br>90.8%
Lack of sufficient studies that evaluated the safety of COVID-19 on pregnant/lactating women	268<br>94.4%
The vaccine may infect the fetus/infant with COVID-19	197<br>69.4%
